# Pancreatic Calcification

**Published:** 2010-08-14

**Authors:** Bilal Mirza

**Affiliations:** Department of Pediatric Surgery, The Children's Hospital and the Institute of Child Health Lahore, Pakistan

An 8-year old girl presented to the emergency room with the complaints of acute epigastric pain and vomiting for two days. Vomiting was non-bilious. There was a past history of recurrent self-limiting similar episodes during the last one year. Patient was prescribed proton pump inhibitors (PPI) and oral suspensions such as sucralfate. There was no history of trauma, rash and jaundice.

General physical examination was unremarkable. There was mild tenderness in the epigastrium on palpation; otherwise abdomen was soft and not distended. Hemoglobin was within normal limits and WBC’s count of 10,500. Random blood glucose, serum electrolytes and renal functions were in normal range. Serum amylase was 113 i.u (Normal &<96 i.u.). An abdominal radiograph was requested that delineated calcifications in the region of pancreas (Fig. 1). 

**Figure:**
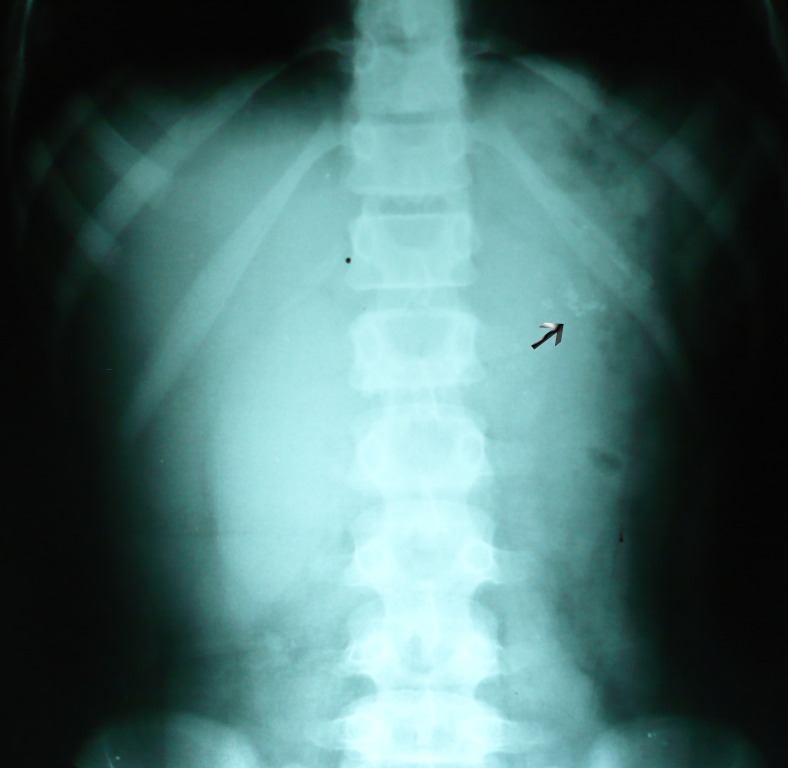
Figure 1: Plain x-ray abdomen showing calcification in the region of pancreas (arrow)

CT scan of abdomen showed a dilated pancreatic duct and calcification in the pancreas and gallstones (Fig. 2). A diagnosis of chronic recurrent pancreatitis with gall stones made and patient provided supportive medical therapy that ameliorated her symptoms. Further workup and possible surgical intervention is planned on her.

**Figure:**
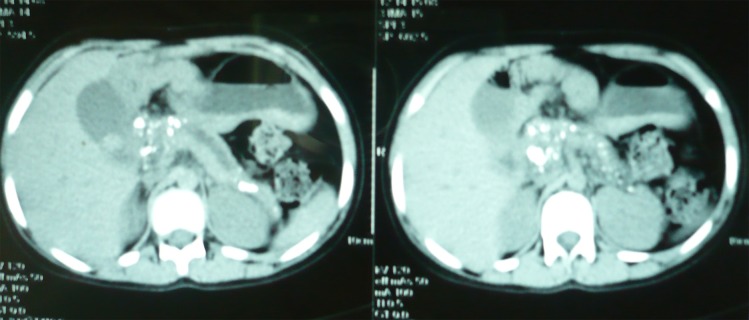
Figure 2: CT scan of abdomen revealing a dilated pancreatic duct, pancreatic calcification and gallstones

## DISCUSSION

Pancreatic calcification is a diagnostic feature of chronic pancreatitis even in the absence of the clinical signs and symptoms. Pancreatic calcification is seen on radiographs in about 30-50% of patients with chronic pancreatitis in adults. Pancreatic calcification is rarely reported in children below ten years, however, its incidence increases after this age [1].

The etiology of the chronic relapsing pancreatitis in children includes; trauma, anatomic abnormalities of the pancreatobiliary ducts, hereditary causes, systemic diseases, choledocholithiasis and cholelithiasis, cystic fibrosis, drugs, inflammatory bowel disease, and infections [1 , 2]. 

Recurrent attacks of the pancreatitis results in progressive damage of the exocrine as well as endocrine functions of the pancreas. Later on, proteinaceous plugs get deposited in the pancreatic ducts and with the passage of time they calcify and can be picked up radiologically. During the relapse of the pancreatitis, patient may present with the features of the acute pancreatitis such as severe epigastric pain and vomiting [3].

In pediatric age group the important cause of the chronic pancreatitis is the congenital anomalies of the pancreas and pancreatobiliary ducts. The important malformations that may present with chronic pancreatitis are choledochal cyst, anomalous union of pancreatobiliary ducts without choledochal cyst, pancreas divisum and annular pancreas. Gallstone disease is another frequently identified cause of the chronic pancreatitis in children. It is usually associated with hemolytic disorders such as thalasemia and hereditary spherocytosis [1 , 2]. 

The anatomical abnormalities of the pancreatobiliary ducts can be diagnosed using modalities like ultrasonography, computed tomography (CT) scan, endoscopic retrograde pancreatography (ERCP) and magnetic resonance pancreatography (MRCP) [1 , 4].

During the relapse of the pancreatitis the supportive management includes nothing by mouth, nasogastric decompression in case of recurrent vomiting, analgesics, PPI, parenteral fluids, antibiotics and nutrition. Some times octreotide infusion has to be instituted in cases of intractable pain [1]. 

The surgical management of chronic relapsing pancreatitis depends upon the etiological factors. In case of gallstones a cholecystectomy may offer relief of the symptoms, however, in case of congenital malformations of the pancreatobiliary systems, major surgical interventions are needed. The procedures may range from simple sphincterotomy and sphincteroplasty to the pancraetectomy and pancreatojejunostomy (Puestow, Duval procedure) or pancreatogastrostomy (Smith procedure). It should be kept under consideration that the mortality and morbidity is high in patients undergoing surgical interventions for chronic pancreatitis [1]. 

## Footnotes

**Source of Support:** Nil

**Conflict of Interest:** None declared

## References

[1] ( 2006). Miyano T. In: Grosfeld JL O’Neill JA Jr, Coran AG, Fonkalsrud EW, Caldamone AA. editors. Pediatric Surgery.

[2] ( 2002). Lesniak RJ, Hohenwalter MD, Taylor AJ. Spectrum of causes of pancreatic calcifications. Am J Radiol.

[3] ( 1970). Rajasuriya K, Thenabadu PN, Leanage RU. Pancreatic calcification following prolonged malnutrition. Am J Dis Child.

[4] ( 2004). Iwao Y, Ritsuko S, Hiroshi O. Imaging: MRCP and ERCP. Jap J Pediatr Surg.

